# Effects of an 8-week multicomponent health-sports program on functional efficiency and volunteering attitudes among university students in Saudi Arabia: a randomized controlled trial

**DOI:** 10.3389/fpubh.2026.1848681

**Published:** 2026-06-16

**Authors:** Ibrahim M. Deyab, Khaled A. Alshdokhi, Ahmed M. A. Abdelbaqy

**Affiliations:** 1 Department of Sports Sciences and Physical Activity, College of Education, University of Ha’il, Ha’il, Saudi Arabia; 2 Department of Physical Education, Faculty of Physical Education, Mansoura University, Mansoura, Egypt

**Keywords:** functional movement, fusion model, multicomponent training, physical fitness, randomized controlled trial, university students, volunteering motivations

## Abstract

**Background:**

Volunteering and regular physical activity are both independently associated with improved physical and psychological wellbeing; however, their combined effects within structured, theory-driven interventions remain insufficiently explored, particularly in the Saudi university context. Multicomponent exercise programs that integrate functional training with brief educational and behavioral components may provide a novel strategy for simultaneously enhancing physical performance and prosocial motivation.

**Objective:**

This study aimed to examine the effects of an 8-week multicomponent exercise program on functional movement quality, physical fitness, and volunteering motivations among university students in Saudi Arabia.

**Methods:**

A randomized controlled trial was conducted with 70 healthy university students (35 males and 35 females; age: 19.3 ± 1.0 years), randomly allocated to either an experimental group (*n* = 35) or a control group (*n* = 35). The experimental group completed an 8-week multicomponent program combining functional training, cardiorespiratory conditioning, and brief health-education sessions linking physical effort to volunteering values, whereas the control group followed a traditional physical education curriculum. Assessments were performed at baseline, mid-intervention (week 4), and post-intervention (week 8). Outcomes included Functional Movement Screen (FMS), physical fitness indicators (50-m sprint, 1,000-m run, sit-and-reach, standing long jump, and vital capacity), and volunteering motivations assessed using the Volunteer Functions Inventory (VFI). Data were analyzed using repeated-measures ANOVA.

**Results:**

No significant baseline differences were observed between the two groups (all *p* > 0.05). After the 8-week intervention, the experimental group demonstrated significantly greater improvements in total Functional Movement Screen (FMS) score (*p* < 0.001, *η*^2^ = 0.18), with notable gains particularly in Active Straight-Leg Raise and Rotary Stability. Significant time × group interaction effects were found for all physical fitness measures: 50-m sprint (*p* = 0.011, *η*^2^ = 0.11), 1,000-m run (*p* = 0.003, *η*^2^ = 0.13), sit-and-reach (*p* = 0.004, *η*^2^ = 0.10), standing long jump (*p* = 0.002, *η*^2^ = 0.12), and vital capacity (*p* = 0.001, *η*^2^ = 0.14). Volunteering motivations also improved substantially in the experimental group, with a mean total VFI increase of 26.0 points (*p* < 0.001, *η*^2^ = 0.16), showing significant gains across all six dimensions (Values, Understanding, Social, Career, Protective, and Enhancement).

**Conclusion:**

An 8-week multicomponent exercise program effectively improves functional movement quality, physical fitness, and volunteering motivations among university students. Integrating functional training with brief educational components appears to enhance both physical and prosocial outcomes, supporting the inclusion of such programs in university physical education and wellness curricula.

## 1 Introduction

Volunteering is widely recognized as a powerful contributor to both individual wellbeing and community development. People who regularly volunteer often experience higher life satisfaction, better psychological health, and stronger social connections than those who do not (1–3). In addition to these benefits, volunteering is associated with personal growth, a greater sense of meaning in life, and improved overall quality of life (4, 5). Longitudinal evidence even suggests that sustained volunteering may encourage healthier lifestyles and support long-term physical wellbeing (6, 7).

The motivations behind volunteering are complex and multidimensional. Classic research has identified six key functions that drive volunteer participation: Values, Understanding, Social, Career, Protective, and Enhancement (8, 9). For university students, volunteering offers an important opportunity to develop soft skills, strengthen social responsibility, and enhance civic engagement—all vital aspects of holistic personal development (10). At a broader level, volunteering helps build social cohesion and community resilience (11, 12).

In Saudi Arabia, volunteering has taken on special importance as part of the Kingdom’s ambitious social and economic transformation under Saudi Vision 2030. National strategies actively encourage volunteer participation to improve quality of life, promote civic engagement, and create healthier communities (13, 14). At the same time, sedentary behavior and low levels of physical activity remain widespread among Saudi university students (15, 16). These parallel challenges highlight the urgent need for innovative university-based interventions capable of addressing both physical health and prosocial engagement simultaneously.

Regular physical activity is a well-established foundation for both physical and psychological health. Among university students, participation in exercise has been linked to better wellbeing, healthier behaviors, improved academic performance, and positive psychosocial outcomes (17–19). Emerging evidence further suggests that physical activity can promote prosocial behavior and greater social engagement (20). This opens promising avenues for interventions that combine structured physical training with value-based educational components.

Among contemporary training approaches, functional training has gained considerable attention because it emphasizes integrated, multi-joint, and multi-planar movements that closely resemble real-life and sporting activities (21, 22). Such programs have been shown to improve movement quality, neuromuscular coordination, balance, muscular fitness, and overall physical performance (23–26). Elements of proprioception and core stability further enhance motor control and movement efficiency in young adults (27–29). The Functional Movement Screen (FMS) has become a standard tool for assessing movement competency and identifying limitations (30–32).

Despite strong evidence supporting the independent benefits of physical activity and volunteering, relatively few studies have examined interventions that deliberately combine functional exercise training with structured volunteering-related educational content. While some research has explored physical activity combined with general health education, randomized controlled trials specifically targeting volunteering motivations through functional training remain limited, particularly in the Saudi context (15, 16).

To address this gap, the present study developed an 8-week multicomponent health-sports program that integrates functional exercise training and cardiorespiratory conditioning with brief, structured educational sessions explicitly linking physical effort to volunteering values and community contribution. The intervention was conceptually grounded in two well-established theoretical frameworks.

The first framework, Experiential Learning Theory (33, 34), suggests that meaningful learning occurs through a cycle of concrete experience, reflective observation, conceptual understanding, and active experimentation. In this program, the physical training sessions offered direct experiential challenges, while the guided reflective discussions helped participants connect feelings of effort, persistence, and teamwork with broader prosocial and volunteering-related values.

The second framework, Self-Determination Theory (SDT) (35, 36), emphasizes that sustained motivation develops when the basic psychological needs of autonomy, competence, and relatedness are satisfied. The program was deliberately designed to support these needs through progressive skill development (competence), active participant involvement in discussions (autonomy), and collaborative group activities (relatedness).

Importantly, the brief health-education modules were specifically crafted to target the six dimensions of the Volunteer Functions Inventory (VFI). For instance, discussions highlighting the societal importance of helping others aimed to strengthen the *Values* function, while prompts encouraging reflection on personal learning and skill development targeted the *Understanding* function. Improvements in physical competence and self-efficacy during training were expected to reinforce these motivational dimensions, creating a synergistic effect between physical and psychosocial outcomes.

Therefore, the purpose of this randomized controlled trial was to examine the effects of an 8-week multicomponent health-sports program on functional movement quality, physical fitness, and volunteering motivations among university students in Saudi Arabia. It was hypothesized that the experimental group would show significantly greater improvements in FMS scores, physical fitness indicators, and VFI dimensions compared to the control group.

## 2 Methods

### 2.1 Study design and participants

This study employed a parallel-group randomized controlled trial design to examine the effects of an 8-week multicomponent exercise program on functional movement quality, physical fitness, and volunteering attitudes among university students. The trial was conducted and reported in full accordance with the CONSORT guidelines to ensure methodological transparency and rigor.

An *a priori* power analysis was performed using G*Power software (version 3.1) to determine the required sample size for detecting a medium effect size (*f* = 0.25) in the time × group interaction term of a repeated-measures ANOVA (*α* = 0.05, power = 0.80, correlation among repeated measures = 0.5). The calculation indicated a minimum of 34 participants. To account for potential attrition and to support secondary analyses, the study recruited 70 healthy university students (35 males and 35 females; mean age 19.3 ± 1.0 years) from the University of Ha’il.

Participants met the following inclusion criteria: age 18–22 years, full-time university enrollment, and no structured physical training in the previous 6 months. Exclusion criteria included any history of musculoskeletal injury that could limit participation, diagnosed cardiovascular or respiratory conditions, or any chronic illness that might compromise safety during exercise. All procedures were approved by the Institutional Research Ethics Committee at the University of Ha’il (Protocol No. H-2025-596) and conducted in accordance with the latest version of the Declaration of Helsinki. Written informed consent was obtained from every participant after a full explanation of the study aims, procedures, potential risks, and benefits. Participants were free to withdraw at any time without consequence.

Throughout the study, participants were instructed to maintain their habitual diet and lifestyle and to refrain from any additional structured exercise outside the assigned program to minimize confounding.

### 2.2 Randomization and blinding

Following baseline assessments, participants were randomly assigned in a 1:1 ratio to either the experimental group or the control group (*n* = 35 per group). Randomization was performed by an independent researcher using a computer-generated random number sequence. Allocation concealment was ensured using sequentially numbered opaque sealed envelopes, which were opened immediately before the first intervention session. This procedure minimized selection bias.

Due to the nature of the exercise interventions, blinding of participants and instructors was not feasible. However, all outcome assessors responsible for conducting the Functional Movement Screen (FMS), physical fitness tests, and Volunteer Functions Inventory (VFI) were kept blinded to group allocation throughout the entire study. The same certified and blinded assessors performed all evaluations at baseline, week 4, and week 8. To maintain blinding integrity, participants were explicitly instructed not to discuss their group assignment or intervention content during assessment sessions. All data were anonymized and coded prior to statistical analysis.

### 2.3 Intervention program

#### 2.3.1 Multicomponent health-sports program (experimental group)

Participants in the experimental group completed an 8-week multicomponent health-sports program consisting of three sessions per week, each lasting approximately 90 min. The program was designed according to contemporary functional training principles, emphasizing integrated multi-joint and multi-planar movement patterns that replicate daily life and sport activities (21, 22). It was further informed by evidence on functional, plyometric, proprioceptive, and core-stability training for improving movement quality, neuromuscular control, balance, and physical fitness (23, 24, 27–29).

Each training session followed a standardized structure:

- *Warm-up (15 min)*: Dynamic mobility and movement preparation exercises to increase neuromuscular activation and joint mobility.
- *Main training phase (65 min)*: Functional strength exercises, agility drills, cardiorespiratory conditioning, balance tasks, and multi-directional movements. Training load and complexity were progressively increased every 2 weeks based on participant adaptation.
- *Cool-down (10 min)*: Low-intensity recovery activities, static stretching, and foam rolling to promote muscular relaxation and mobility.

#### 2.3.2 Health-education module

A structured 5-min educational module was delivered immediately after the warm-up in every training session. This module aimed to explicitly link physical activity participation with volunteering-related values, including teamwork, cooperation, social responsibility, persistence, and community engagement (4, 10, 20).

The module followed a standardized format consisting of three components:

1. *Brief Didactic Discussion (2 min)*: The instructor provided interactive explanations connecting physical effort, challenge, and teamwork during exercise with prosocial values and volunteering.
2. *Reflective Discussion Prompt (2 min)*: Participants discussed open-ended questions in pairs or small groups to reflect on concepts such as helping others, cooperation, and community contribution.
3. *Action Commitment (1 min)*: Each participant identified and verbally committed to one specific voluntary prosocial action they intended to perform before the next session.

To ensure fidelity and consistency, the same certified instructor delivered all modules using a pre-prepared standardized script across all 24 sessions. Intervention fidelity was monitored by a research assistant through direct observation during weeks 3 and 6, with no major deviations from the protocol identified.

The overall structure of the multicomponent health-sports program, including the integration of the health-education module, is summarized in Table 1.

**Table 1:** Overview of the multicomponent health-sports program structure.

Training component	Primary functional focus	Prosocial/educational theme
Dynamic mobility & warm up	Mobility, balance, movement preparation	Readiness to support others and adaptability
Agility & coordination training	Agility, coordination, footwork	Teamwork, cooperation, and attentiveness
Functional strength & stability	Core stability, unilateral strength	Responsibility, persistence, and reliability
Explosive power development	Power production and dynamic balance	Continuous self-improvement and positive effort
Multi planar functional movement	Integrated movement control	Adaptability and rapid response in community settings
Recovery & flexibility	Mobility recovery	Mindfulness, sustainability, and self-care

The program consisted of 3 sessions per week (90 min each) over 8 weeks. Each session included a 15-min warm-up, 65-min main training phase, 10-min cool-down, and a 5-min health-education module. Training load and exercise complexity were progressively increased every 2 weeks according to participants’ adaptation and performance.

#### 2.3.3 Control group (traditional physical education program)

Participants in the control group followed a conventional university physical education curriculum matched for duration (8 weeks), frequency (3 sessions/week), and session length (90 min). Exercise intensity was kept comparable to minimize differences in overall workload.

The control program consisted primarily of traditional resistance exercises, continuous aerobic activities, and basic sport drills. Unlike the experimental group, it did not emphasize functional multi-planar movements, core stabilization, or dynamic balance (21, 22). Importantly, the control condition did not include any structured health-education, reflective discussions, or volunteering-related content.

Intervention adherence was high, with participants in the experimental group attending an average of 92.4% of scheduled sessions. No participants withdrew from the study.

### 2.4 Outcome measures

All assessments were conducted at baseline (week 0), mid-intervention (week 4), and post-intervention (week 8).

#### 2.4.1 Functional movement assessment

Functional movement quality was evaluated using the Functional Movement Screen (FMS), a standardized tool designed to identify limitations and asymmetries in fundamental movement patterns (30). The FMS comprises seven tests (overhead squat, hurdle step, in-line lunge, shoulder mobility, active straight-leg raise, trunk stability push-up, and rotary stability). Each test is scored on a 0–3 ordinal scale (0 = pain, 1 = unable to complete, 2 = completed with compensation, 3 = perfect execution). The total score ranges from 0 to 21. All assessments were performed by a single FMS Level 2 certified examiner. Pilot reliability testing on 10 volunteers yielded excellent intra-rater (ICC = 0.93) and inter-rater (ICC = 0.90) reliability.

#### 2.4.2 Physical fitness assessment

Physical fitness was assessed using five standardized field tests aligned with national student health standards:

1. 50-m sprint (speed)
2. 1,000-m run (cardiorespiratory endurance)
3. Sit-and-reach test (flexibility)
4. Standing long jump (explosive power)
5. Vital capacity (spirometry)

#### 2.4.3 Volunteering motivations assessment

Volunteering motivations were measured using the Volunteer Functions Inventory (VFI) (9), a 30-item validated questionnaire assessing six dimensions: Values, Understanding, Social, Career, Protective, and Enhancement. The Arabic version was translated using forward-backward method and showed high internal consistency (Cronbach’s *α* = 0.89).

### 2.5 Statistical analysis

All statistical analyses were performed using IBM SPSS Statistics version 26.0 (IBM Corp., Armonk, NY, USA). Data normality was checked using the Shapiro–Wilk test, and homogeneity of variances was examined using Levene’s test. Baseline differences between groups were assessed with independent-samples t-tests.

The main analyses consisted of two-way repeated-measures ANOVA, with time (baseline, week 4, and week 8) as the within-subject factor and group (experimental vs. control) as the between-subject factor. Mauchly’s test was used to evaluate the sphericity assumption, and Greenhouse–Geisser corrections were applied when this assumption was violated. Significant time × group interactions were followed by Bonferroni-adjusted *post-hoc* comparisons. Partial eta squared (*η*^2^) was calculated to determine effect sizes, interpreted as small (0.01), medium (0.06), or large (0.14). Statistical significance was set at *p* < 0.05 (two-tailed).

All analyses were conducted according to the intention-to-treat principle. No participants were lost to follow-up, and there were no missing data for any outcome variable across the three assessment points.

## 3 Results

**Figure 1:**
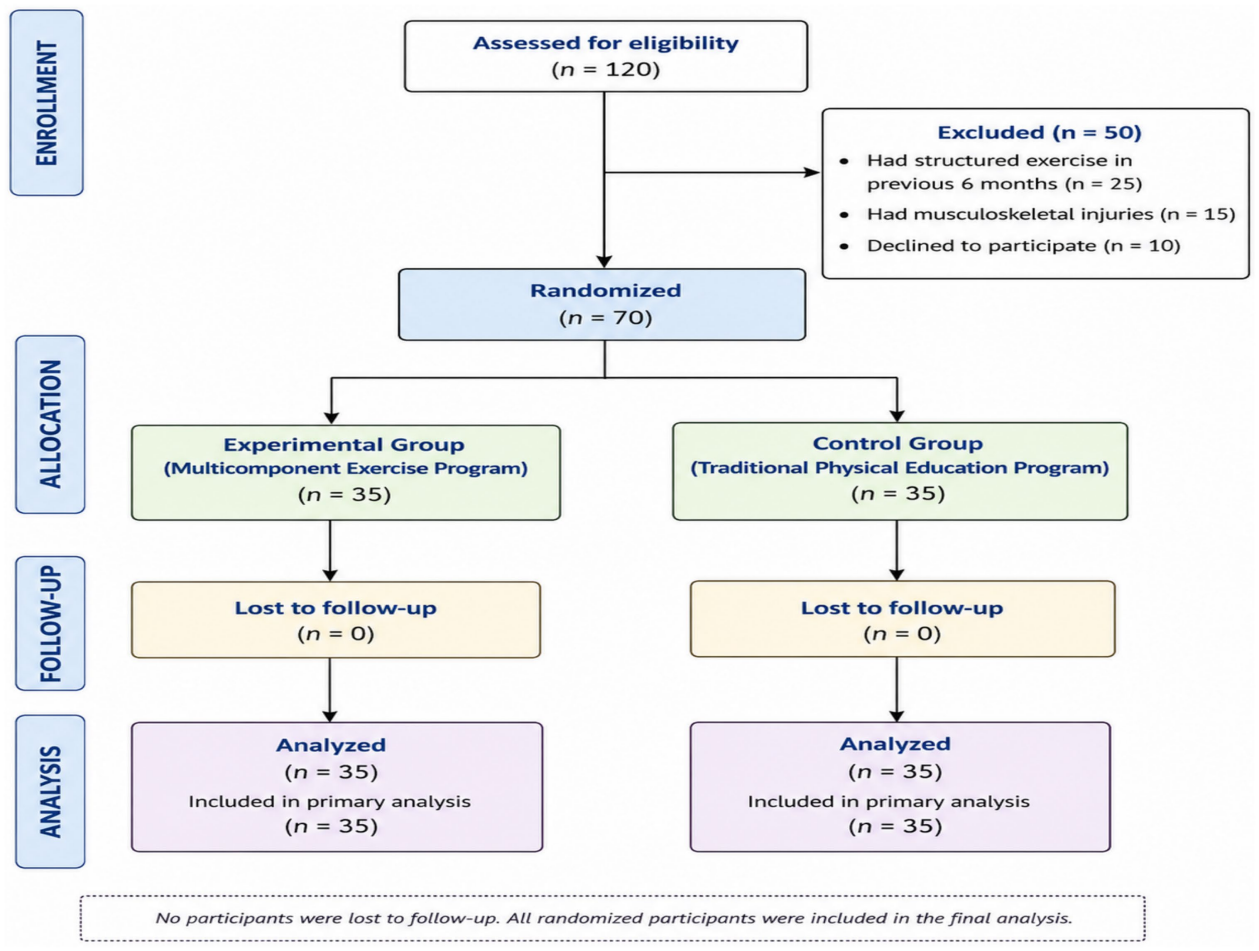
CONSORT flow diagram of participant recruitment, randomization, follow-up, and analysis. No participants were lost to follow-up. All randomized participants were included in the final analysis according to the intention-to-treat principle.

All 70 participants completed the study with no dropouts or missing data. Baseline characteristics were comparable between groups (Table 2), and no significant between-group differences were observed in any outcome variable at baseline (all *p* > 0.05), indicating successful randomization and baseline equivalence (see Figure 1).

**Table 2:** Baseline characteristics of participants.

Variable	Control group (*n* = 35)	Experimental group (*n* = 35)	*p*-value
Age (years)	19.4 ± 1.1	19.3 ± 0.9	0.812
Height (cm)	168.7 ± 7.2	169.1 ± 8.1	0.874
Body mass (kg)	64.8 ± 9.3	65.2 ± 10.4	0.891

Data are shown as mean ± standard deviation. There were no significant differences between the two groups at the start (*p* > 0.05), which confirms that the randomization worked and the groups were comparable.

### 3.1 Functional movement screen (FMS)

#### 3.1.1 Baseline equivalence

Independent-samples t-tests revealed no significant differences between the experimental and control groups in any individual Functional Movement Screen (FMS) component or total FMS score at baseline (all *p* > 0.05).

#### 3.1.2 Changes over time

The 8-week multicomponent intervention produced substantial improvements in functional movement quality in the experimental group, whereas the control group demonstrated only modest changes over time.

Repeated-measures ANOVA demonstrated significant time × group interaction effects for several FMS components, including In-line Lunge (*F*(1, 68) = 4.36, *p* = 0.039, *η*^2^ = 0.10), Active Straight-Leg Raise (*F*(1, 68) = 7.21, *p* = 0.008, *η*^2^ = 0.14), Trunk Stability Push-up (*F*(1, 68) = 6.35, *p* = 0.012, *η*^2^ = 0.12), and Rotary Stability (*F*(1, 68) = 8.02, *p* = 0.006, *η*^2^ = 0.15). No significant interaction effect was observed for Shoulder Mobility (*p* > 0.05). Bonferroni-adjusted *post-hoc* comparisons indicated significantly greater improvements from baseline to week 8 in the experimental group for the significant FMS components (all adjusted *p* < 0.05).

**Figure 2:**
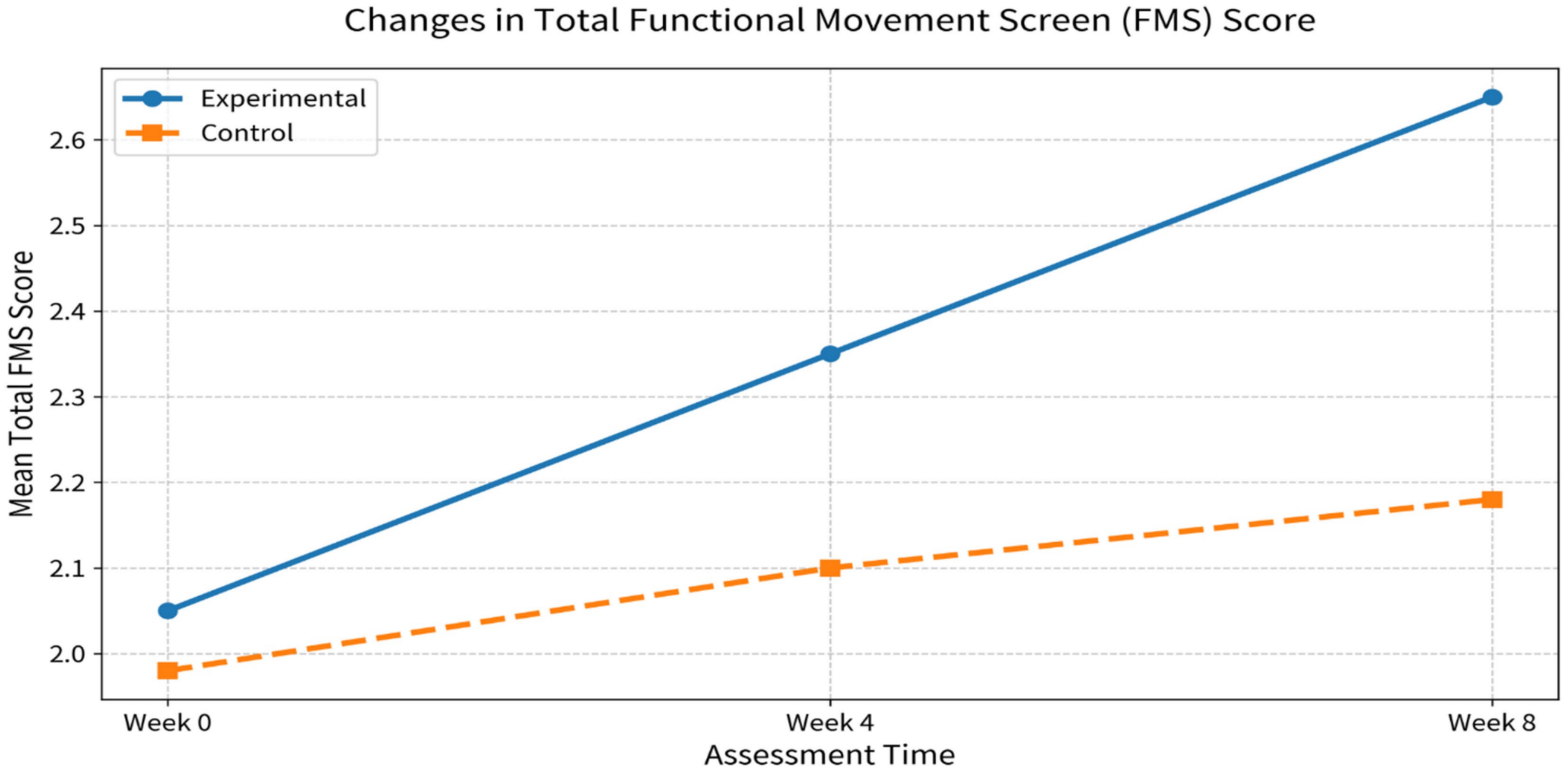
Changes in total Functional Movement Screen (FMS) score across the 8-week intervention period. Values are presented as mean. The experimental group demonstrated consistent and progressive improvement compared to the control group.

Figure 2 shows the changes in total FMS score across the three assessment points.

Detailed temporal changes in individual FMS components are presented in Tables 3, 4.

**Table 3:** Temporal changes in FMS component scores (mean ± SD).

Test	Group	Week 0	Week 4	Week 8	*F* (df)	*p*	*η* ^2^	95% CI
Overhead squat	Experimental	2.32 ± 0.61	2.55 ± 0.50	2.78 ± 0.42	**F(1, 68) = 2.41**	0.102	0.05	[−0.12, 0.68]
	Control	2.18 ± 0.68	2.30 ± 0.63	2.40 ± 0.60				
Hurdle step	Experimental	2.05 ± 0.58	2.28 ± 0.52	2.52 ± 0.47	**F(1, 68) = 2.12**	0.118	0.04	[−0.08, 0.52]
	Control	1.98 ± 0.62	2.10 ± 0.60	2.20 ± 0.58				
In-line lunge	Experimental	2.12 ± 0.54	2.48 ± 0.49	2.80 ± 0.36	**F(1, 68) = 4.36**	0.039*	**0.10**	[0.04, 0.89]
	Control	2.01 ± 0.57	2.12 ± 0.55	2.18 ± 0.51				

Bold values indicate statistically significant time × group interaction effects (*p* < 0.05). Degrees of freedom are (1, 68) for all *F*-tests.

**Table 4:** Changes in key FMS components with largest improvements (Week 0 to Week 8).

Test	Group	Week 0	Week 8	*F* (df)	*p*	*η* ^2^	95% CI
Active straight-leg raise	Experimental	2.02 ± 0.52	2.76 ± 0.38	**F(1, 68) = 7.21**	0.008*	**0.14**	[0.22, 0.98]
	Control	1.92 ± 0.50	2.10 ± 0.47				
Trunk Stability push-up	Experimental	1.98 ± 0.63	2.62 ± 0.44	**F(1, 68) = 6.3**5	0.012*	**0.12**	[0.18, 0.86]
	Control	1.91 ± 0.58	2.18 ± 0.49				
Rotary stability	Experimental	1.85 ± 0.57	2.58 ± 0.41	**F(1, 68) = 8.0**2	0.006*	**0.15**	[0.31, 1.05]
	Control	1.82 ± 0.52	2.12 ± 0.45				

Bold values indicate statistically significant time × group interaction effects (*p* < 0.05).

### 3.2 Physical fitness outcomes

#### 3.2.1 Baseline comparison

Before the intervention, no significant between-group differences were observed in any physical fitness variable at baseline (all *p* > 0.05), indicating comparable fitness levels between groups prior to the intervention.

#### 3.2.2 Post-intervention changes

After 8 weeks, the experimental group demonstrated clear improvements across all physical fitness measures, whereas the control group showed only modest changes. Repeated-measures ANOVA revealed significant time × group interaction effects for all assessed variables, including 50-m sprint (*F*(1, 68) = 6.91, *p* = 0.011, *η*^2^ = 0.11), 1,000-m run (*F*(1, 68) = 7.22, *p* = 0.008, *η*^2^ = 0.13), sit-and-reach (*F*(1, 68) = 5.84, *p* = 0.016, *η*^2^ = 0.10), standing long jump (*F*(1, 68) = 6.35, *p* = 0.012, *η*^2^ = 0.12), and vital capacity (*F*(1, 68) = 7.10, *p* = 0.009, *η*^2^ = 0.14). Bonferroni-adjusted *post-hoc* comparisons confirmed that improvements from baseline to week 8 were significantly greater in the experimental group across all physical fitness outcomes (all adjusted *p* < 0.05).

### 3.3 Volunteering motivations (VFI)

**Figure 3:**
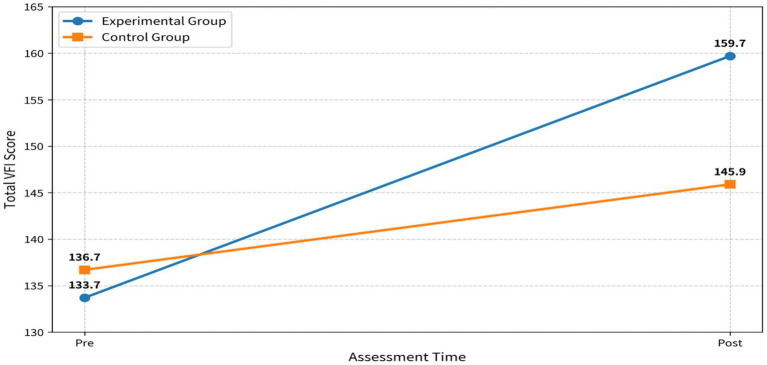
Changes in Volunteer Functions Inventory (VFI) dimensions from pre- to post-intervention. Values are presented as mean scores. The experimental group (blue) showed consistently greater improvements compared to the control group (orange).

The 8-week multicomponent health-sports intervention resulted in substantial improvements in volunteering-related motivations in the experimental group, whereas the control group demonstrated only modest changes (see Figure 3).

Repeated-measures ANOVA indicated a significant time × group interaction effect for the total Volunteer Functions Inventory (VFI) score (*F*(1, 68) = 9.35, *p* = 0.003, *η*^2^ = 0.16). The experimental group demonstrated a mean increase of 26.0 points, compared with 9.2 points in the control group. Bonferroni-adjusted *post-hoc* comparisons confirmed significantly greater improvements in the experimental group across the total VFI score and all VFI dimensions (all adjusted *p* < 0.05).

Analysis of the individual VFI dimensions demonstrated significant improvements across all six subscales, with the largest effect sizes observed in the Values (*η*^2^ = 0.14) and Understanding (*η*^2^ = 0.12) dimensions. Significant improvements were also identified in the Social, Enhancement, Career, and Protective dimensions.

Collectively, the findings demonstrate that the multicomponent intervention produced consistent improvements across both physical and psychosocial domains, supporting the integrated design of the program.

## 4 Discussion

The present randomized controlled trial demonstrated that an 8-week multicomponent health-sports program, which combined functional training, cardiorespiratory conditioning, and brief structured educational sessions on volunteering values, produced significantly greater improvements in functional movement quality, physical fitness, and volunteering motivations compared to a traditional physical education curriculum among Saudi university students. The experimental group showed meaningful gains across most Functional Movement Screen (FMS) components, all physical fitness measures, and all six dimensions of the Volunteer Functions Inventory (VFI). These results highlight the promising value of integrating physical training with psychosocial elements in university health-promotion programs. To our knowledge, this study is among the first randomized controlled trials in Saudi Arabia to simultaneously investigate physical performance and volunteering-related motivations in university students (15, 16).

### 4.1 Functional movement adaptations

The superior improvements in functional movement quality observed in the experimental group can largely be attributed to the integrated, multi-planar nature of the training program. Unlike traditional resistance exercises, functional training emphasizes coordinated, real-life movement patterns that engage multiple muscle groups across different planes of motion. This approach appears to enhance neuromuscular coordination, proprioception, and dynamic postural control more effectively (21, 22, 29).

Particularly notable gains were seen in the Active Straight-Leg Raise, Trunk Stability Push-up, and Rotary Stability tests. These findings align well with previous studies showing that core stability and proprioceptive training can improve lumbo-pelvic control and overall movement competency (24, 27, 28). On the other hand, Shoulder Mobility and Overhead Squat did not show statistically significant interaction effects. This is likely due to the principle of training specificity, suggesting that more targeted mobility work may be needed to address certain movement limitations (22, 31).

### 4.2 Physical fitness improvements

The intervention led to significant improvements across all physical fitness tests, indicating that the program was both physiologically effective and well tolerated by the students. The combination of agility drills, functional strength exercises, plyometric movements, and cardiorespiratory conditioning appears to have enhanced neuromuscular efficiency, muscular power, flexibility, and aerobic capacity (23, 25, 26).

Gains in 50-m sprint and standing long jump likely reflect improved lower-body power and neuromuscular activation, while better performance in the 1,000-m run and increased vital capacity point to enhanced cardiovascular and respiratory efficiency (21, 28, 37). The high adherence rate (92.4%) further suggests strong acceptability and feasibility of the program within the university setting. The inclusion of brief reflective educational discussions may have also played a role in maintaining participant motivation and engagement throughout the 8 weeks (20).

### 4.3 Volunteering motivations and prosocial outcomes

One of the most notable findings was the substantial improvement across all VFI dimensions in the experimental group, with the largest effect sizes observed in the Values and Understanding domains. This suggests that participants developed stronger motivational orientations toward volunteering and a deeper appreciation of its personal and social value. Improvements in the Social and Enhancement dimensions also indicate increased feelings of connectedness and positive self-perception (see Tables 5, 6).

**Table 5:** Changes in physical fitness variables (mean ± SD).

Variable	Group	Week 0	Week 8	*F* (df)	*p*	*η* ^2^	95% CI
50-m sprint (s)	Experimental	7.42 ± 0.41	7.08 ± 0.28	*F*(1, 68) = 6.91	0.011*	0.11	[0.08, 0.42]
	Control	7.46 ± 0.52	7.34 ± 0.48				
1,000-m run (min)	Experimental	4.11 ± 0.60	3.72 ± 0.52	F(1, 68) = 7.22	0.008*	0.13	[0.11, 0.58]
	Control	4.13 ± 0.55	3.98 ± 0.54				
Sit-and-reach (cm)	Experimental	13.6 ± 5.3	18.4 ± 4.8	*F*(1, 68) = 5.84	0.016*	0.10	[1.2, 7.8]
	Control	13.4 ± 4.9	14.2 ± 4.7				
Standing long jump (cm)	Experimental	218 ± 15	232 ± 12	*F*(1, 68) = 6.35	0.012*	0.12	[5, 23]
	Control	217 ± 18	223 ± 17				
Vital capacity (mL)	Experimental	4,520 ± 740	5,400 ± 690	*F*(1, 68) = 7.10	0.009*	0.14	[310, 1,050]
	Control	4,460 ± 760	4,550 ± 750				

Values are presented as mean ± standard deviation. Significant time × group interaction effects are indicated by * (*p* < 0.05). Bonferroni-adjusted *post-hoc* analyses demonstrated significantly greater improvements in the experimental group compared with the control group across all physical fitness outcomes.

**Table 6:** Changes in Volunteer Functions Inventory (VFI) dimensions and total score (mean ± SD).

Dimension	Group	Pre (mean ± SD)	Post (mean ± SD)	*F* (df)	*p*	*η* ^2^	95% CI
Values	Experimental	24.6 ± 4.2	30.8 ± 3.9	*F*(1, 68) = 8.12	0.006*	0.14	[3.2, 8.9]
	Control	25.1 ± 4.5	27.0 ± 4.2				
Understanding	Experimental	23.9 ± 4.0	29.7 ± 3.8	*F*(1, 68) = 7.54	0.008*	0.12	[2.8, 7.9]
	Control	24.5 ± 4.3	26.1 ± 4.0				
Social	Experimental	22.8 ± 3.7	26.9 ± 3.5	*F*(1, 68) = 6.43	0.013*	0.10	[1.5, 5.8]
	Control	23.2 ± 4.1	24.8 ± 3.9				
Career	Experimental	20.7 ± 3.9	23.6 ± 3.6	*F*(1, 68) = 5.21	0.025*	0.08	[0.9, 4.2]
	Control	21.3 ± 4.2	22.5 ± 3.8				
Protective	Experimental	19.8 ± 3.8	22.6 ± 3.5	*F*(1, 68) = 5.04	0.028*	0.07	[0.7, 3.9]
	Control	20.2 ± 4.0	21.5 ± 3.7				
Enhancement	Experimental	21.9 ± 3.6	26.1 ± 3.4	*F*(1, 68) = 6.88	0.010*	0.11	[1.8, 5.6]
	Control	22.4 ± 3.9	24.0 ± 3.6				
Total VFI	Experimental	133.7 ± 14.8	159.7 ± 13.9	*F*(1, 68) = 9.35	0.003*	0.16	[14.2, 37.8]
	Control	136.7 ± 15.2	145.9 ± 14.6				

Values are presented as mean ± standard deviation. Significant time × group interaction effects are indicated by * (*p* < 0.05). Bonferroni-adjusted post-hoc analyses confirmed significantly greater improvements in the experimental group across all VFI dimensions and total score.

It is important to note, however, that the Volunteer Functions Inventory measures motivational orientations rather than actual volunteering behavior (9). Therefore, these results reflect positive changes in prosocial dispositions and readiness to volunteer, rather than confirmed increases in real-world volunteering activity. Future longitudinal studies should examine whether these motivational gains are sustained and eventually translate into actual civic engagement.

### 4.4 Theoretical interpretation

The observed improvements can be meaningfully understood through the complementary lenses of Experiential Learning Theory and Self-Determination Theory (SDT). Experiential Learning Theory posits that deep learning occurs through cycles of concrete experience, reflection, conceptualization, and active experimentation (33, 34). In this program, the physical training provided rich experiential challenges, while the structured reflective discussions helped students connect effort, persistence, and teamwork with broader prosocial and volunteering values.

In parallel, Self-Determination Theory emphasizes the role of autonomy, competence, and relatedness in fostering internalized motivation (35, 36). The progressive skill development, collaborative activities, and reflective goal-setting in the program likely supported these basic psychological needs, facilitating the internalization of volunteering-related values.

### 4.5 A key mechanism

Underlying the transfer from improved physical performance to increased volunteering motivation seems to be the enhancement of self-efficacy. Through repeated mastery of challenging functional movements and persistence during demanding training sessions, participants likely developed stronger beliefs in their own capabilities. According to Bandura’s Social Cognitive Theory, such mastery experiences are among the most powerful sources of self-efficacy (40). This increased sense of competence may have generalized beyond the physical domain, leading students to think: “If I can handle these difficult workouts and see real progress, then I am also capable of contributing meaningfully to my community through volunteering.” This generalization helps explain how a physical training program can positively influence prosocial motivations.

### 4.6 Practical implications

These findings support the integration of functional training with brief value-based educational modules within university physical education programs. Such multicomponent approaches offer a practical and scalable way to address both physical inactivity and low volunteering engagement among young adults. This model aligns closely with the objectives of Saudi Vision 2030, which prioritizes healthier lifestyles and greater civic participation (13, 14). Universities may benefit from adopting similar programs to develop students who are both physically competent and socially responsible.

### 4.7 Comparison with previous literature

The results are consistent with previous research on functional and multicomponent training programs and their positive effects on movement quality and physical fitness (23–25). The present study extends this literature by incorporating structured volunteering-value education within the exercise sessions and examining its impact on volunteering motivations in a Saudi university context.

Compared with recent studies that combined health education and exercise (41), the current trial used a robust randomized controlled design and focused specifically on volunteering-related motivational outcomes, providing additional insights relevant to the Saudi setting.

These findings are also consistent with previous evidence highlighting the positive influence of integrated exercise and psychosocial interventions on health-related and behavioral outcomes among university students (38, 39).

### 4.8 Study limitations and future directions

Several limitations should be acknowledged when interpreting the findings of this study. First, participants were recruited from a single university in Saudi Arabia, which restricts the generalizability of the results to other student populations, educational institutions, or different cultural contexts. Second, although adherence to the supervised training sessions was high, we did not objectively monitor participants’ physical activity levels or dietary habits outside the program, so these variables may have been subject to self-report bias.

Third, and perhaps most importantly, the intervention lasted only 8 weeks and did not include any long-term follow-up assessments. While we observed meaningful improvements in both physical fitness and volunteering motivations immediately after the program, the durability of these changes—particularly the psychosocial ones—remains uncertain. Motivational orientations can be relatively transient following short-term interventions, and it is unclear whether the gains in VFI scores will persist over time or translate into sustained real-world volunteering behavior and civic engagement. Future programs might benefit from incorporating booster sessions to help maintain these motivational improvements.

Future research should address these limitations by employing multi-center designs with larger and more diverse samples, using objective measures of physical activity (such as accelerometers), and including longer follow-up periods (e.g., 3 or 6 months post-intervention). It would be especially valuable to include behavioral indicators of actual volunteering participation rather than relying solely on self-reported motivational measures.

Overall, despite these limitations, the present study provides encouraging preliminary evidence that integrating functional exercise training with structured volunteering-value education can simultaneously enhance physical performance and psychosocial outcomes among university students.

## 5 Conclusion

In conclusion, this randomized controlled trial demonstrated that an 8-week multicomponent health-sports program integrating functional training, cardiorespiratory conditioning, and structured volunteering-value education produced significantly greater improvements in functional movement quality, physical fitness, and volunteering motivations compared to a traditional physical education curriculum among Saudi university students.

The program’s unique strength lies in its ability to simultaneously enhance physical performance and multiple dimensions of volunteering motivation through the integration of brief, structured reflective discussions that connect physical effort with prosocial values. These findings highlight the value of moving beyond siloed physical training toward more holistic, theory-driven interventions in higher education.

Within the Saudi context, this model aligns closely with the objectives of Saudi Vision 2030 by supporting both improved population health and increased civic engagement among young adults. The intervention offers universities a feasible, acceptable, and scalable framework that can be implemented within existing physical education programs to foster physically competent and socially responsible graduates.

Future studies should examine the long-term sustainability of these effects, assess whether improvements in volunteering motivations lead to actual volunteering behavior, and evaluate the program’s effectiveness across diverse populations and settings.

## Data Availability

The raw data supporting the conclusions of this article will be made available by the authors, without undue reservation.
